# Editorial: Sensors and sensor systems for detection of emerging environmental contaminants

**DOI:** 10.3389/fchem.2022.1100402

**Published:** 2022-12-01

**Authors:** Alisa Rudnitskaya, Larisa Lvova

**Affiliations:** ^1^ Department of Chemistry and CESAM, University of Aveiro, Aveiro, Portugal; ^2^ Department of Chemical Science and Technologies, University of Rome “Tor Vergata”, Rome, Italy

**Keywords:** (bio)chemical sensors, sensor arrays: electronic noses, electronic tongues, environmental analysis, emerging contaminants

It is a great opportunity for us to organize the important Research Topic “Sensors and Sensor Systems for Detection of Emerging Environmental Contaminants” that highlights recent developments in sensor applications in environmental analysis.

In recent years, growth in human activities and occurrence of natural events related to the climate change have led to the release of contaminants causing significant impact on the environment. In this context, there is a growing interest in *in-situ* and real time detection of contaminants present in water, air, and soil. Sensors for gas and liquid analysis are attractive tools for fast screening of specific contaminants in the environment due to their low cost, easy automation, and possibility of *in-situ* applications.

This Research Topic collects three articles on the development of novel sensors for the detection of environmentally relevant analytes and one research paper on the application of an electronic nose in robotics.

Development of novel sensing materials is of paramount importance for successful sensor applications in environmental analysis. In the paper of Beduk et al. the modification of laser-scribed graphene with molecularly imprinted polymer afforded a sensitive and selective sensor for bisphenol A (BPA) at the environmentally relevant concentration levels. Integration of the sensor with portable measuring platform enables its application in on-site BPA monitoring tasks.

Other carbonaceous nanostructured materials, the carbon dots (CDs), have been employed by Wang et al. for the selective detection of ions and bioimaging. CDs is a novel nanomaterial gaining popularity in sensing applications due to its easy synthesis and functionalization, low cost, good water dispersibility and photostability. However, practical applications of CDs are often limited by their relatively low quantum yield. CDs doped with P have demonstrated high fluorescence quantum yield in aqueous solutions with selective and sensitive quenching response toward Fe^3+^ and MnO_4_^−^ ions.

Contrary to bisphenol A, fluoride, iron (III) and permanganate that are known for their toxicity, hydrogen peroxide is not considered to be harmful to the environment due to its low stability. However, the development of the robust sensors with low detection limits for H_2_O_2_ detection is of interest for environmental applications as they are widely used as transducers in enzymatic biosensors. In work of Chen et al., a nanocomposite of graphene oxide and phenothiazine dye, new methylene blue, was used as a sensitive layer in electrochemical sensor for the determination of hydrogen peroxide. High sensitivity and low detection limit exhibited by the proposed sensor make it promising for direct H_2_O_2_ detection as well as biosensor development.

Electronic noses are gaining acceptance as valuable tool for on-site environmental monitoring. When employed in the open sampling systems, electronic noses are exposed to uncontrolled environment. One of the important tasks of the electronic noses in this scenario is detection of appearance of new, potentially harmful, chemical compounds or sudden increase of the concentration of one of the compounds. Successful detection of unknown analyte requires the use of unsupervised learning techniques for signal processing of electronic noses. One of such methods is an ensemble learning based approach (ELBA), that integrates several one-class classifiers and learns online. Reported by Fan et al., the ELBA algorithm has allowed the detection of gas exposures at the same time recognizing suspect short-term sensor baseline drifts. The proposed classifier can be easily integrated with supervised learning models when the prior knowledge of target analytes is partially available.

